# Structure of the complex of C1q-like 3 protein with adhesion-GPCR BAI3

**DOI:** 10.1038/s42003-025-08112-w

**Published:** 2025-05-03

**Authors:** Yi Miao, Haoqing Wang, Kevin M. Jude, Jie Wang, Jinzhao Wang, Marius Wernig, Thomas C. Südhof

**Affiliations:** 1 Department of Molecular and Cellular Physiology, Stanford University School of Medicine, Stanford, CA USA; 2 Howard Hughes Medical Institute, Stanford University School of Medicine, Stanford, CA USA; 3 Sarafan CHEM-H, Stanford University, Stanford, CA USA; 4 Department of Structural Biology, Stanford University School of Medicine, Stanford, CA USA; 5 Institute for Stem Cell Biology and Regenerative Medicine, Stanford University School of Medicine, Stanford, CA USA; 6 Department of Pathology, Stanford University School of Medicine, Stanford, CA USA; 7 Present Address: Division of Life Science, The Hong Kong University of Science and Technology, Hong Kong, China

**Keywords:** Cryoelectron microscopy, Molecular neuroscience

## Abstract

The adhesion-GPCR Brain-specific Angiogenesis Inhibitor-3 (BAI3) plays a crucial role in organizing synapses in the brain. However, how BAI3 engages one of its ligands, the C1q-like proteins (C1qls), remains largely unexplored. Here, we present the single-particle cryo-electron microscopy (cryo-EM) structure of the C1ql3-BAI3 complex at 2.8 Å resolution. The structure reveals a hexameric configuration, where C1ql3 forms a central homotrimer that effectively captures three BAI3 molecules. These BAI3 molecules fit snugly into the grooves between the trimeric C1q domains of the C1qls, employing calcium ion (Ca^2+^)-mediated interactions that differ from previously characterized structures of C1q-like domain-mediated complexes. Furthermore, we conducted mutant analysis and cell surface staining, which confirmed the essential contact residues involved in this interaction. This unique binding mechanism not only enhances our understanding of the C1ql-BAI3-mediated synaptic organization but also sheds light on the functional dynamics of BAI3 in the brain.

## Abstract (web summary)

This study reports the structural characterization of C1q-like protein 3 (C1ql3) with adhesion-GPCR brain-specific angiogenesis inhibitor-3 (BAI3).

## Introduction

BAIs are a family of adhesion-GPCRs comprising BAI1, BAI2 and BAI3 that are implicated in multiple developmental functions^1–3^. The function of BAIs is thought to be mediated by their ligands, secreted C1qls and GPI-anchored Reticulon-4 receptors (RTN4Rs, a.k.a. NoGo receptors)^4–9^. The importance of C1ql-binding to BAIs in synaptic connections has been revealed from C1ql1 and BAI3 deletion studies showing severely reduced climbing-fiber synapse formation in the cerebellum^4,5,8,9^ and dramatically impaired synapses formed by accessory olfactory nucleus (AON) neurons onto olfactory bulb granule cells^8^. C1qls utilize the globular trimeric C1q-like domain to engage BAI3^7^, yet the mode of the C1ql-BAI3 interaction has not been structurally resolved. Although many mammalian proteins with diverse functions contain C1q-like domains similar to those of C1qls, only a single C1q-like domain complex structure with surface receptor has been structurally characterized. This complex of the hexameric Cerebellin-1 (Cbln1) C1q-like domain with dimeric N-terminal domain (NTD) of GluD2^10^ serves as a paradigm for C1q-like domain interactions, but if C1q-like domains engage other partners in a similar manner is not known. C1qls are thought to form large 18-meric assemblies^11^, suggesting a higher oligomeric state of the complex and a potentially different interaction mode. We set out to elucidate the atomic structure of BAI3 with C1qls.

## Results and discussion

**Fig. 1:**
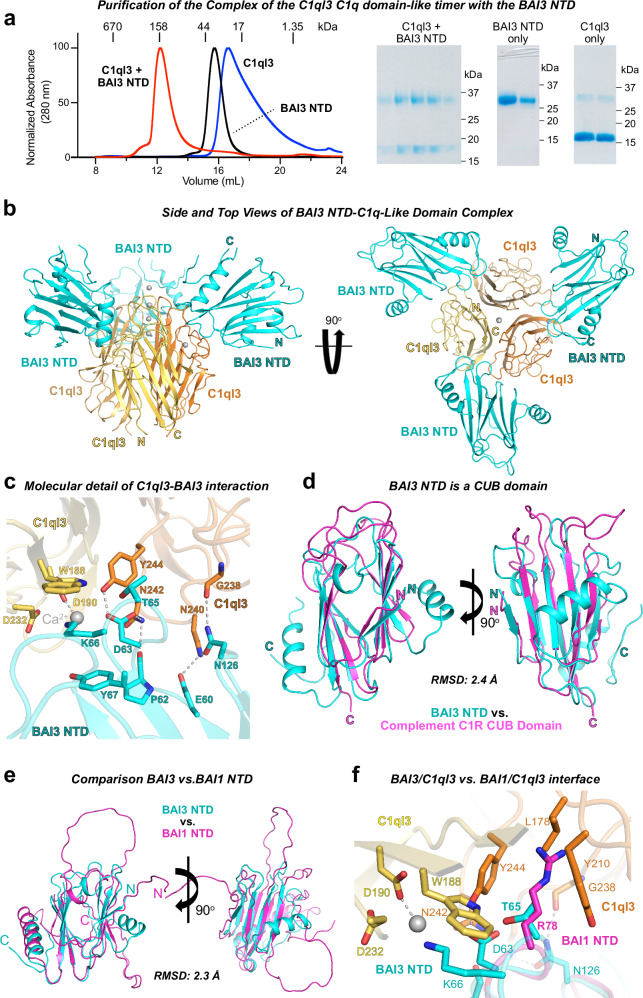
The atomic structure of BAI3 N-terminal domains bound to the C1q-like domain trimers of C1ql3 reveals that BAI3 N-terminal domains represent an atypical CUB domain that nestles into the asymmetric interfaces between two C1q-like domains. **a** The isolated BAI3 NTD (residues 26–290) forms a tight stoichiometric complex with the C1q-like domain trimer of C1ql3 (residues 122-255), as monitored by size-exclusion chromatography (left) and Coomassie-stained SDS-PAGE gels (right). **b** Overall structure of C1q-like domain trimer from C1ql3 (colored in different shades of orange) in a complex with BAI3 NTDs (cyan). Ca^2+^-ions are shown as gray spheres, and selected N- and C-termini are identified. **c** Molecular determinants of BAI3 NTD (cyan) and C1ql3 C1q-like domain (orange) interactions. Ca^2+^-ions are shown as gray spheres and interacting residues as sticks, while hydrogen bonds or salt bridges are indicated by dashed gray lines. **d** Structure superposition of the BAI3 NTD (cyan) with the C1R CUB domain (magenta; top hit ranked by DALI search). **e** Structure superposition of mouse Alpha Fold predicted BAI1 NTD (light magenta) to BAI3 NTD (cyan). **f** Detailed C1ql3-BAI NTD interaction with Alpha Fold predicted BAI1 NTD (light magenta) superposed to BAI3 NTD (cyan). T65 mutation from BAI3 to R78 in BAI1 leads to steric clashes with interacting C1ql3.

The extracellular sequence of BAI3 is composed of an NTD, four thrombospondin type-1 repeats, a hormone-binding domain (that doesn’t bind hormones), and a GAIN domain^12^. We first confirmed that the BAI3 NTD forms a stable complex with C1ql3 globular C1q-like domain as reported earlier^5^ (Fig. 1a). We then determined the structure of the mouse C1ql3-BAI3 NTD complex by single-particle cryoEM to a resolution of 2.8 Å (Fig. 1b; Supplementary Fig. 1). At the core of the complex, three globular C1q-like domains form a trimeric quaternary structure with four Ca^2+^-ions bound at the trimeric symmetry axis consistent with structures of the isolated C1q-like domain^11^ (Fig. 1b). Three BAI3 NTDs bind to the C1ql3 trimer (Fig. 1b). Each BAI3 NTD interacts with the C1ql3 trimer at a composite interface between two C1ql3 molecules, creating a heterohexamer (Fig. 1c).

Within the C1ql3-BAI3 interface (Fig. 1c; Supplementary Fig. 2a), one C1ql3 copy establishes a Ca^2+^-mediated interaction with the BAI3 NTD. Significantly, the Ca^2+^-ion is coordinated by both the C1ql3 C1q-like domain and the BAI3 NTD. Asp180 and Asp232 of the C1q-like domain direct the coordination of the Ca^2+^-ion, while Lys66 and Asp63 of the BAI3 NTD participate in the water-mediated coordination of the Ca^2+^-ion. Although these water molecules are integral to the Ca^2+^-ion coordination, they were not visible in the electric potential map and so were not modeled. Furthermore, C1ql3 Trp188, BAI3 Lys66, and BAI3 Tyr67 engage in a π-stacking interaction that further stabilizes the complex. The second C1q-like domain in the binding interface also forms extensive interactions with the BAI3 NTD, where C1ql3 Tyr244 and Asn242 engage in hydrogen bonds with BAI3 Asp63, while C1ql3 Asn240 forms a hydrogen bond with BAI3 Glu60 (Fig. 1c). In summary, each BAI3 molecule forms a Ca^2+^-mediated interface with the C1q-like domains of C1ql3 that is characterized by extensive intermolecular interactions and spans a total of ~564 Å^2^.

The cryoEM structure also allowed us to identify the domain type of the BAI3 NTD. DALI searches^13^ uncovered top hits that were CUB domains from various proteins, notably the CUB domain from the serine protease C1r of the complement activation C1 complex^14^ (Fig. 1d). Despite low amino acid identities ranging from 8% to 19%, the root mean square deviation (RMSD) values for the top ten hits ranged from only 1.9 to 3.2 Å. Superposition of the BAI3 NTD onto the C1r CUB domain highlights a conserved core of β strands, with the primary distinction being that the BAI3 NTD contains additional α helices outside the core β sheet that are not usually present in CUB domains and that may mediate additional, as yet unidentified interactions by the NTD. Alpha Fold^15^ predicted the core β strands with high precision (Supplementary Fig. 2b) but failed to model the extensive loop regions where Alpha Fold has a low prediction confidence. Recently, the N-terminal CUB domain of another adhesion GPCR, GPR126, has been shown to have a regulatory function in aGPCR signaling^16^. While our structural studies do not provide insights into the signaling potency of the BAI3 CUB domain, the binding of the ligand C1ql3 to this domain suggests that BAI3 NTD CUB domain might have signaling and/or regulatory function. It’s important to note that the GPR126 CUB domain differs from the BAI3 CUB domain in size (~109 vs. ~170 amino acids) and structure, lacking the additional α helices and loop regions present in BAI3.

We sought to understand why BAI2 and BIA3 bind to C1ql, whereas BAI1 does not, despite very similar sequences. To assess whether the NTDs of BAI1 and BAI2 may adopt a similar structure as the BAI3 NTD and to model their interactions with C1ql C1q-like domains, we aligned the NTD sequences of mouse BAI1, BAI2, and BAI3 (Supplementary Fig. 2c). Despite sharing only ~30% sequence identity, the residues crucial for C1ql recognition are conserved in BAI2 but not in BAI1. Since Alpha Fold predicts the BAI3 NTD core structure reasonably well, we further modeled the BAI1 and BAI2 NTD into the C1ql3-BAI3 complex structure. Consistent with the sequence alignment, BAI2 shows comparable binding to C1ql3 while BAI1 does not (Supplementary Fig. 2e, 2f). Notably, the Thr65Arg substitution from BAI3 to BAI1 results in steric clashes with the C1ql3 C1q-like domain (Fig. 1e, f). Additionally, the Glu60Arg substitution in BAI1 disrupts the hydrogen bond connection with C1ql3 Asn240. These results indicate that all BAI NTDs adopt a similar conformation resembling CUB domains with additional structural loop elements but that only BAI2 and BAI3, and not BAI1 can bind C1qls. In contrast to the lack of conservation of the C1ql-binding residues in BAI1 compared to BAI2 and BAI3, the BAI-binding residues of C1ql3 are fully conserved in the three other C1ql isoforms (C1ql1, C1ql2, and C1ql4; Supplementary Fig. 2d) consistent with earlier studies^7^.

Next, we examined the evolution of BAI NTDs by aligning the BAI NTD sequences from zebrafish, bovine, rat, mouse, and human (Supplementary Fig. 2g). The C1ql-interacting loops were highly conserved among BAI2 and BAI3 orthologs but key residues were consistently substituted in BAI1 orthologs, specifically Glu60 and Thr65, indicating a shared absence of BAI1 binding to C1qls across species (Supplementary Fig. 2g). Additionally, the BAI2 NTD exhibited a conserved negatively charged region not present in BAI1 and BAI3. In summary, the evolutionary divergence of BAIs is consistent with their differential ligand binding properties and functions.

To further characterize the binding of C1ql3 to BAI receptors, we expressed BAI3 and BAI1 on the surface of HEK293T cells and performed staining with C1ql3 (Supplementary Fig. 3a and Supplementary Fig. 4). Our results confirm that C1ql3 binds to BAI3 but not to BAI1. A single residue substitution of BAI3 Thr65 to Arg disrupts C1ql3 binding. However, reversing the BAI1 loop to match BAI3 does not restore C1ql3 binding, suggesting that overall domain folding and additional residues may also influence binding. At the C1ql3-BAI3 interface, mutations of BAI3 residues Lys66 and Tyr67 to Ala disrupt C1ql3 binding. Conversely, single mutations of C1ql3, such as Asp190Ala or Tyr244Ala, retain binding to BAI3, while a triple mutant (Asn240Ala, Asn242Ala and Tyr244Ala) disrupts this interaction (Supplementary Fig. 3b, c). Overall, these results indicate that specific residues at both the BAI3 and C1ql3 interfaces are critical for their interaction.

**Fig. 2:**
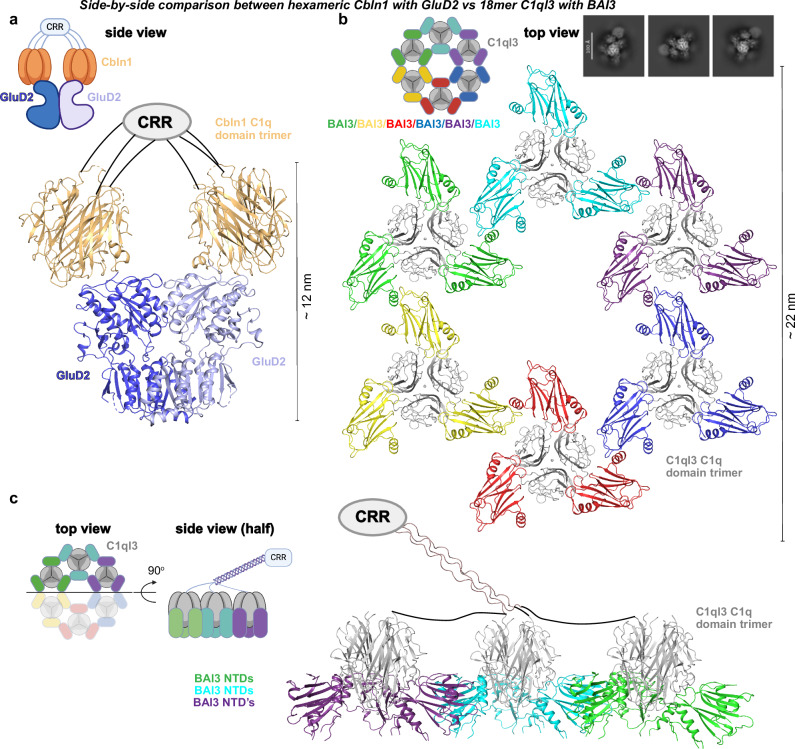
Structural comparison between Cbln1-GluD2 with C1ql3-BAI3 reveals radically distinct binding mode and stoichiometry. **a** Side view of the hexametric Cbln1 with dimeric GluD2. Cbln1 are shown in orange and GluD2 dimer is colored dark and light blue (PDB: 5KCA). **b** Top view of the proposed model of the 36-meric C1ql3-BAI3 NTD super-complex. C1ql3’s are shown in gray, BAI3 NTDs are colored differentially as indicated. A few representative 2D class averages of neighboring C1ql3-BAI3 NTD hexamer are shown in the figure. Scale bar in the 2D class averages represent 100 Å. **c** Split side view of the proposed model of the 36-meric C1ql3-BAI3 NTD super-complex.

Finally, we aimed to understand whether C1q-like domains exhibit differential recognition modes for its receptors. To date, the structure of only one C1q-like domain complex with a receptor has been reported, namely that of the synaptic organizer Cbln1 with its receptor GluD2^10^. Surprisingly, despite the high structural homology between of C1ql3 and Cbln1, the binding mode and binding stoichiometry of the BAI3 NTD to the C1ql3 C1q domain differs from that of the Cbln1 C1q-like domain to the GluD2 NTD (Fig. 2). The trimeric C1q-like domains of Cbln1 bind to one copy of the GluD2 NTD, allowing hexameric Cbln1 to bind to the dimeric GluD2 NTD in a 3:1 stoichiometry (Fig. 2a). Additionally, the C1q trimeric C1q domain has been structurally characterized with a nanobody and the Fc region of IgG, both showing a 3:1 stoichiometry^17,18^. In contrast, the trimeric C1q-like domains of C1ql3 bind to three copies of BAI3 in a 3:3 stoichiometry (Fig. 1b). Another striking difference between Cbln1 and C1ql3 is the involvement of Ca^2+^-ion. Four Ca^2+^-ions are present at the trimeric symmetry axis of C1ql3 (Fig. 1b), stabilizing the trimer as previous reported^11^. Additionally, Ca^2+^-ions bound on the trimer surface directly contribute to C1ql3-BAI3 interaction (Fig. 1c). Thus, the trimeric C1q-like domains of Cbln1 and C1ql3 utilize different surface areas for binding to their targets, GluD2 and BAI3.

In contrast to hexameric Cbln1, C1ql3 potentially forms an 18-mer structure^11^, consisting of a dimeric cysteine-rich region (CRR) and a trimeric collagen-like domain, followed by a trimeric C1q domain. Given this structural arrangement (Fig. 1b) and 2D classifications (Supplementary Fig. 1a), we hypothesize that C1ql3 and BAI3 may form a hexagonal lattice (Fig. 2b, c). This hexagonal lattice is proposed analogous to that observed for the tumor necrosis factor (TNF) ligand and receptor families^19^. Overall, this suggests that the synaptic organizers of the C1ql family have the potential to assemble BAI aGPCRs at the synapse into larger clusters. Ligand-induced oligomerization of BAI aGPCRs could play an important regulatory role in GPCR signaling and may contribute to the mechanism by which C1ql3 supports synapse formation^20^. This offers new insights into the multifunctional roles of C1ql proteins in neuronal development. We would like to emphasize that the concept of a hexagonal lattice remains a hypothesis. Further investigations are needed to fully understand this intriguing ligand-receptor pair.

In summary, we report the structure of the complex of the C1ql3 C1q-like domain with the BAI3 NTD, demonstrating a previously not observed geometry and binding interface for a C1q-like domain/receptor complex and suggesting that C1q domains are versatile binding domains that may have a panoply of functional interactions. Moreover, our findings indicate that C1qls may cluster synaptic BAI adhesion-GPCRs, implying a specific mechanism for C1qls as synaptic organizers and highlighting a novel aspect of adhesion-GPCR function.

## Methods

### Protein expression

The C1ql3 constructs were cloned into the pEG BacMam vectors and expressed in Expi293F™ GnTI^-^ cells, following the manufacturer’s recommended protocol (Thermo Fisher Scientific). Expi293 cells were cultured to a density of 3 × 10^6^ cells/mL. DNA was introduced into the Expi293 cells using 2.7 μL of GIBCO^TM^ ExpiFectamineTM 293 Reagent per mL of cells. Enhancers were incorporated 18 h post-transfection, and cells were subsequently incubated for either 48 or 72 h before harvesting. The purification of the expressed proteins was carried out through Ni^2+^-NTA affinity column chromatography, followed by size exclusion chromatography (SEC) using 1X HBS buffer (10 mM HEPES, pH 7.2, 150 mM NaCl) in the presence of 2 mM CaCl_2_. For cell surface staining experiments, C1ql3 and its mutant were cloned with HA tag into pEG BacMam vectors and expressed in Expi293F™ cells, the purification procedure is identical as described above.

The Mouse BAI3 N-terminal domain (NTD) encompassing residues 26–290 was cloned into the pVLAD8L vector, as previously described^21^, which encodes a 3 C protease cleavage site, an Fc-tag and a C-terminal His tag following BAI3 NTD. The Bacmam virus is packaged as previously described^21^. HEK293S GnTI^−^ cells were grown to a cell density of 2 × 10^6^ cells per mL before infecting with the BacMam virus. A final concentration of 5 mM sodium butyrate was applied to the cells. The HEK293 GnTI^−^ cell line was generously provided by Prof. H.G. Khorana’s lab at the Massachusetts Institute of Technology, Cambridge, MA, USA. Cell supernatant was harvested 48-hour post-infection, and the BAI3 NTD protein was purified by Ni^2+^-NTA affinity column chromatography. Fc tag was removed by 3 C protease cleavage, and BAI3 NTD was further purified though size exclusion chromatography in 1 X HBS buffer.

### Cryo-electron microscopy sample preparation and data collection

To prepared C1ql3-BAI3 samples for cryo-EM analysis, proteins were mixed at 1: 1.1 molar ratio, followed by size exclusion chromatography in 1x HBS buffer with 2 mM CaCl_2_. Fractions corresponding to the complex were collected and concentrated. Aliquots of 3.2 μL of 0.7 mg/mL complex in the presence of 0.01% Fluorinated Octyl Maltoside were applied to glow-discharged 300 mesh UltraAuFoil® (1.2/1.3) or Quantifoil® (1.2/1.3) grids. The grids were blotted for 3 seconds using a Vitrobot Mark IV and plunged into liquid ethane. Grids were screened by a 200 kV Glacios microscope (Thermo Fisher Scientific) before collection at 300 kV Krios microscope (Thermo Fisher Scientific). Data were collected at a magnification corresponding to a 0.653 Å per physical pixel. The dose was set to a total of 50 electrons per Å^2^. Data collection was performed using SerialEM^22^ with a nominal defocus range set from −0.8 to −2.4 μm. 3 total datasets were collected, including 2 datasets using UltraAuFoil and Quantifoil without stage tilt and 1 dataset using Quantifoil and 30-degree stage tilt. All data merged were collected on the same microscope.

### Image processing

All image processing was performed in cryoSPARC^23^. Movies were motion corrected using patch motion correction followed by patch contrast transfer functions (CTFs) correction. Notably, motion correction and CTF correction were performed separately in cryoSPARC for the three datasets. The initial particles were picked using Topaz picker^24^. Multiple rounds of reference-free 2D classification were performed for each dataset individually, and then to generate an intermediate stack of 502,102 particles. These particles were then used in ab initio reconstruction into three classes. Duplicate particles were removed to generate a final stack of 167,880 particles, which was refined by non-uniform refinement and local refinement^25^. This resulted in a 2.8 Å reconstruction of the C1ql3-BAI3 NTD complex (Supplementary Fig. 1).

### Model building and refinement for cryoEM

The C1ql3^11^ and BAI3 Alpha Fold^15^ models were docked into the map using UCSF Chimera^26^. The resultant model was then manually built in Coot^27^, followed by automated refinement using Phenix real space refine^28^. The detailed statistics were shown in Table 1.

**Table1:** Cryo-EM data collection, refinement, and validation statistics

	C1ql3-BAI3 NTD (EMDB-43605) (PDB 8VWY)
Data collection and processing	
Magnification	130,000
Voltage (kV)	300
Electron exposure (e–/Å^2^)	50
Defocus range (μm)	−0.8 to −2.4
Pixel size (Å)	0.653
Symmetry imposed	C3
Initial particle images (no.)	6,958,616
Final particle images (no.)	167,880
Map resolution (Å)	2.8
FSC threshold	0.143
Map resolution range (Å)	2.6–3.4
Refinement	
Initial model used (PDB code)	4QQH, Alpha Fold
Model resolution (Å)	3.1
FSC threshold	0.5
Map sharpening *B* factor (Å^2^)	−128.7
Model composition	
Non-hydrogen atoms	6293
Protein residues	825
Ligands	7
*B* factors (Å^2^)	
Protein	67.81
Ligand	56.74
R.m.s. deviations	
Bond lengths (Å)	0.006
Bond angles (°)	0.579
Validation	
MolProbity score	2.54
Clashscore	11.76
Poor rotamers (%)	6.10
Ramachandran plot	
Favored (%)	94.76
Allowed (%)	5.24
Disallowed (%)	0

### Cell surface staining

The cell surface staining experiments were performed similarly as previously described^6^. Briefly, HEK293T cells were transfected with 0.625 μg total DNA (0.125 μg mCherry + 0.5 μg FLAG-tagged BAI expression plasmid). After 24 h, 100 nM final concentration of HA tatgged C1ql3 protein or mutant was added in DMEM media and cells were incubated at room temperature for 1 hr. Cells were washed once in PBS prior to fixation using 4% PFA/4% sucrose/PBS for 20 min at 4°. Fixed cells were stained with primary antibody (Rabbit Anti-FLAG Antibody, Sigma, F7425 and Mouse anti-HA Antibody, Covance, MMS101R) and secondary antibody (Goat anti-Mouse IgG (H + L) Highly Cross-Adsorbed Secondary Antibody, Alexa Fluor™ 488, Thermo Fisher Scientific, A-11029 and Goat anti-Rabbit IgG (H + L) Highly Cross-Adsorbed Secondary Antibody, Alexa Fluor™ 647, Thermo Fisher Scientific, A-21245). Cells were mounted on microscope slides and images were recorded using Nikon confocal microscope. Final image analysis and figure processing were performed using Fiji software.

### Statistics and reproducibility

No statistics analysis was performed in this manuscript. CryoEM refinement and data validation statistics were reported from cryoSPARC and PHENIX^23,28^. Raw data for cryoEM was deposited to https://www.ebi.ac.uk/empiar/ and could be utilized to reproduce the structural characterizations.

### Reporting summary

Further information on research design is available in the Nature Portfolio Reporting Summary linked to this article.

## Supplementary information

Supplementary Information

Supplementary Data 1

Description of Additional Supplementary Files

Reporting Summary

## Data Availability

The structures have been deposited into Electron Microscopy Data Bank under the accession code 43605 and Protein Data Bank under the accession code 8VWY. Raw data are deposited to https://www.ebi.ac.uk/empiar/ under accession code 12083. The raw data for size exclusion chromatography is provided in Supplementary Data 1. All other data is deposited in Stanford Data Repository https://purl.stanford.edu/qm670gq0127. The information will be publicly available as of the date of publication.
