# Supplementary material for: Structure of the complex of C1q-like 3 protein with adhesion-GPCR BAI3
Source: Commun Biol. 2025 May 3;8:693. doi: 10.1038/s42003-025-08112-w (PMC12048575; doi:10.1038/s42003-025-08112-w)
Supplement: Supplementary file 1 — Supplementary Information [file 42003_2025_8112_MOESM1_ESM.pdf]

Supplementary Information

Structure of the complex of C1q-like 3 protein with adhesion-GPCR BAI3

Yi Miao[1,2,7,*], Haoqing Wang[3], Kevin M. Jude[1,2,4], Jie Wang[1,7], Jinzhao Wang[1,5,6], Marius Wernig[5,6], and Thomas C. Südhof[1,2,*]

[1]Department of Molecular and Cellular Physiology, Stanford University School of Medicine, Stanford, California 94305, USA

[2]Howard Hughes Medical Institute, Stanford University School of Medicine, Stanford, California 94305, USA

[3]Sarafan CHEM-H, Stanford University, Stanford, CA 94305, USA

[4]Department of Structural Biology, Stanford University School of Medicine, Stanford, California 94305, USA

[5] Institute for Stem Cell Biology and Regenerative Medicine, Stanford University School of Medicine, Stanford, CA 94305, USA

[6]Department of Pathology, Stanford University School of Medicine, Stanford, CA 94305, USA

[7]Current Address: Division of Life Science, The Hong Kong University of Science and Technology, Hong Kong, China.

*Correspondence: yimiao@ust.hk (Y.M.); tcs1@stanford.edu (T.C.S.)

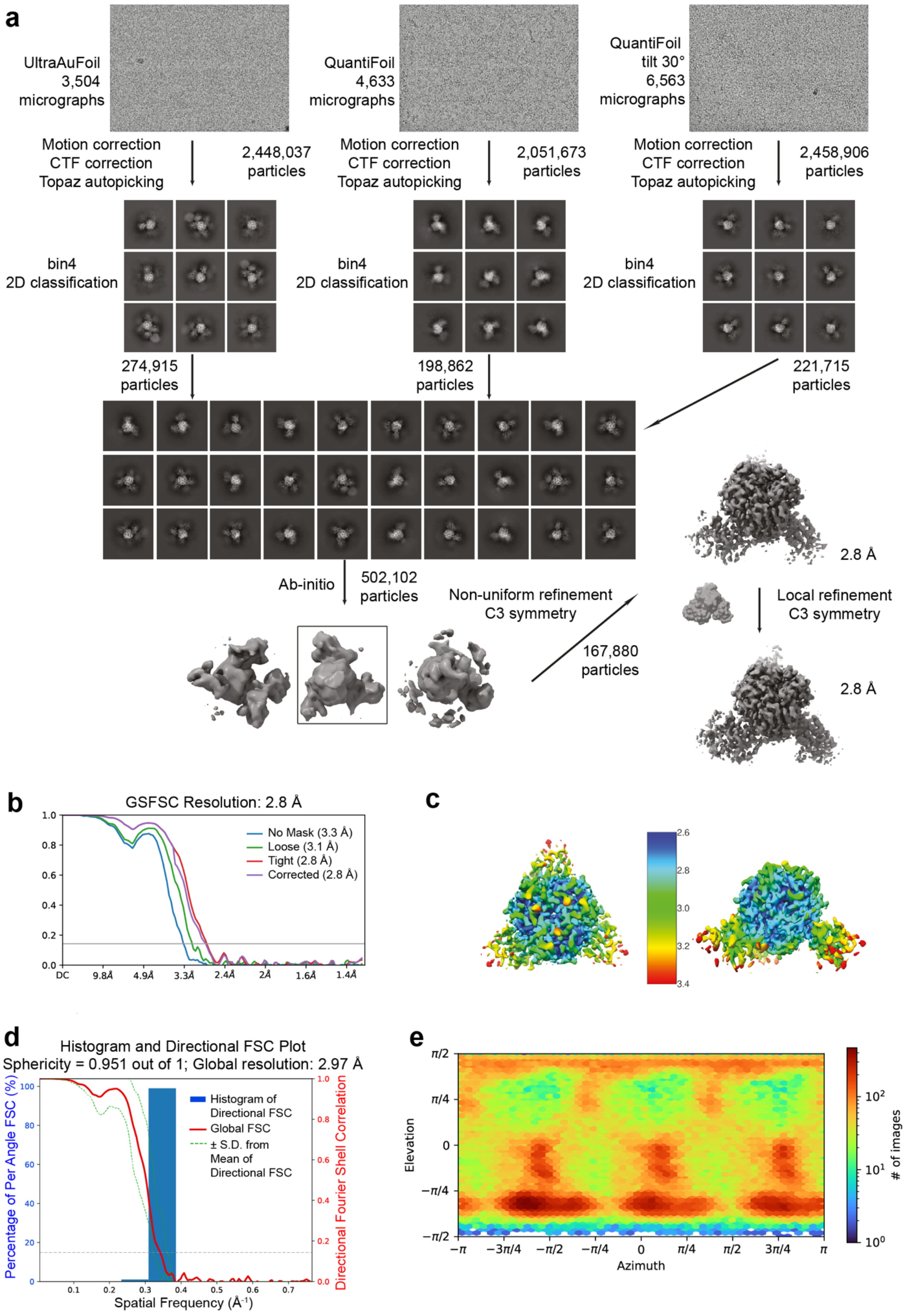
a
UltraAuFoil
3,504
micrographs
QuantiFoil
4,633
micrographs
QuantiFoil
tilt 30°
6,563
micrographs
Motion correction
CTF correction
Topaz autopicking
2,448,037
particles
2,051,673
particles
2,458,906
particles
bin4
2D classification
274,915
particles
198,862
particles
221,715
particles
Ab-initio
502,102
particles
Non-uniform refinement
C3 symmetry
167,880
particles
2.8 Å
Local refinement
C3 symmetry
2.8 Å
b
GSFSC Resolution: 2.8 Å
No Mask (3.3 Å)
Loose (3.1 Å)
Tight (2.8 Å)
Corrected (2.8 Å)
DC
9.8Å
4.9Å
3.3Å
2.4Å
2Å
1.6Å
1.4Å
c
2.6
2.8
3.0
3.2
3.4
d
Histogram and Directional FSC Plot
Sphericity = 0.951 out of 1; Global resolution: 2.97 Å
Percentage of Per Angle FSC (%)
Directional Fourier Shell Correlation
Histogram of Directional FSC
Global FSC
± S.D. from Mean of Directional FSC
Spatial Frequency (Å-1)
e
Elevation
Azimuth
# of images

Supplementary Figure 1: Cryo-EM data processing of C1ql3-BAI3 NTD complex

a. Cryo-EM image processing workflow for C1ql3-BAI3 NTD complex. Please note that a few representative 2D class averages also shown in Fig. 2, these could be highly similar or identical to 2D class averages here. Due to data merging and the nature of the 2D averages, some 2D class averages in this figure are highly similar or identical.

b. Fourier shell correlation (FSC) curve with the estimated resolution for non-uniform refinement, the resolution is calculated at FSC = 0.143.

c. Local resolution map for C1ql3-BAI3 NTD complex viewed from two directions.

d. Histogram and directional FSC plot.

e. Angular distribution of C1ql3-BAI3 NTD complex.

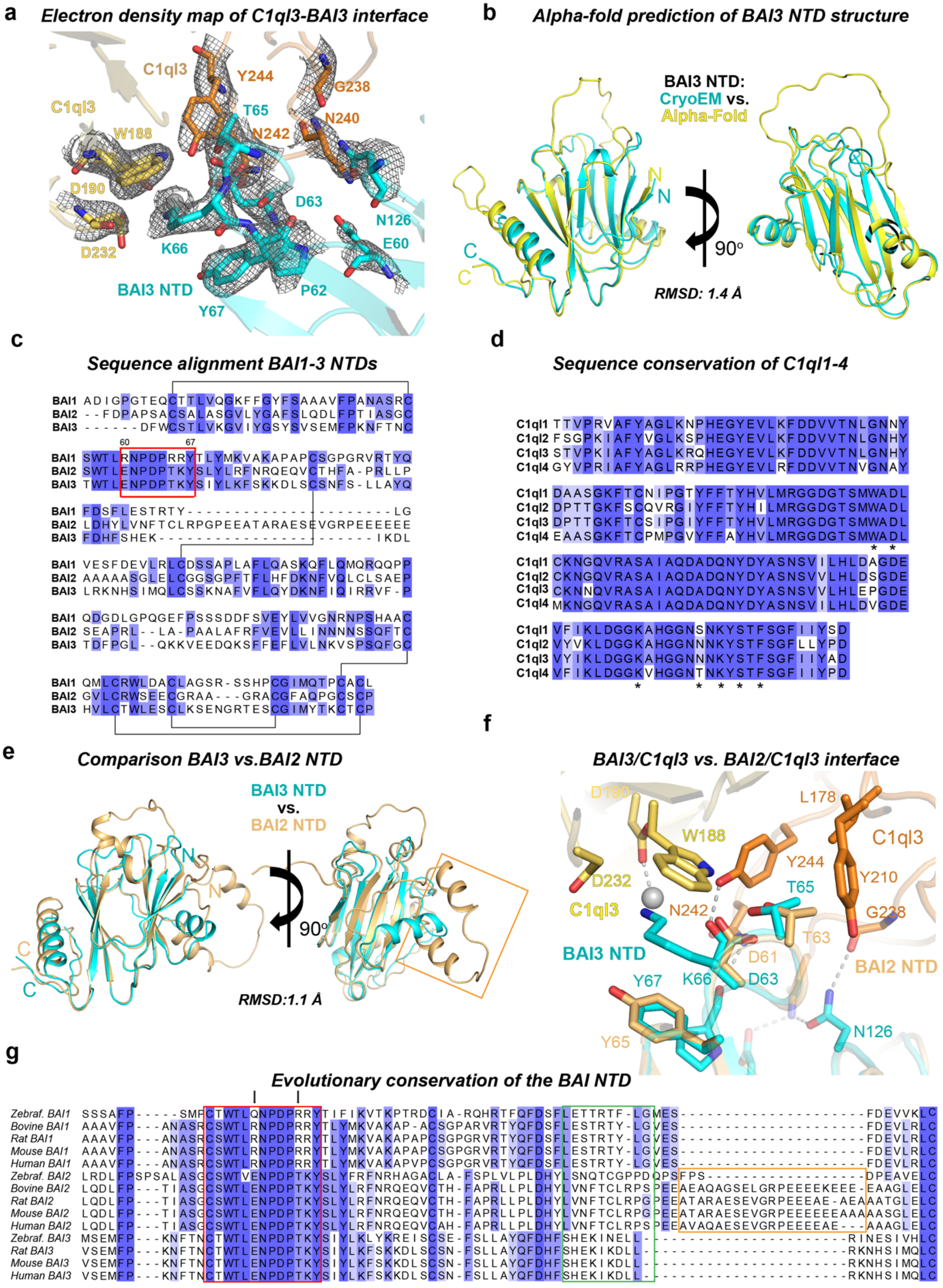
a
Electron density map of C1ql3-BAI3 interface
C1ql3
C1ql3
Y244
G238
T65
N240
N242
W188
D190
D63
N126
E60
D232
K66
BAI3 NTD
P62
Y67
b
Alpha-fold prediction of BAI3 NTD structure
BAI3 NTD:
CryoEM vs.
Alpha-Fold
N
N
C
C
90°
RMSD: 1.4 Å
c
Sequence alignment BAI1-3 NTDs
BAI1 ADIGPGTEQCTTLVQGKFFGYFSAAAVFPANASRC
BAI2 --FDPAPSACSALASGVLYGAFSLQDLFPTIASGC
BAI3 ------DFWCSTLVKGVIYGSYSVSEMFPKNFTNC
60 67
BAI1 SWTLRNPDPRRYTLYMKVAKAPAPCSGPGRVRTYQ
BAI2 SWTLENPDPTKYSLYLRFNRQEQVCTHFA-PRLLP
BAI3 TWTLENPDPTKYSIYLKFSKKDLSCSNFS-LLAYQ
BAI1 FDSFLESTRTY-------------------------LG
BAI2 LDHYLVNFTCLRPGPEEATARAESEVGRPEEEEEEE
BAI3 FDHFSHEK---------------------------IKDL
BAI1 VESFDEVLRLCDSSAPLAFLQASKQFLQMQRQQPP
BAI2 AAAAASGLELCGGSGPFTFLHFDKNFVQLCLSAEP
BAI3 LRKNHSIMQLCSSKNAFVFLQYDKNFIQIRRVF-P
BAI1 QDGDLGPQGEFPSSSDDFSVEYLVVGNRNPSHAAC
BAI2 SEAPRL--LA-PAALAFRFVEVLLINNNNSSQFTC
BAI3 TDFPGL--QKKVEEDQKSFFEFLVLNKVSPSQFGC
BAI1 QMLCRWLDACLAGSR-SSHPCGIMQTPCACL
BAI2 GVLCRWSEECGRAA---GRACGFAQPGCSCP
BAI3 HVLCTWLESCLKSENGRTESCGIMYTKCTCP
d
Sequence conservation of C1ql1-4
C1ql1 TTVPRVAFYAGLKNPHEGYEVLKFDDVVTNLGNNY
C1ql2 FSGPKIAFYVGLKSPHEGYEVLKFDDVVTNLGNHY
C1ql3 STVPKIAFYAGLKRQHEGYEVLKFDDVVTNLGNHY
C1ql4 GYVPRIAFYAGLRRPHEGYEVLRFDDVVTNVGNAY
C1ql1 DAASGKFTCNIPGTYFFTYHVLMRGGDGTSMWADL
C1ql2 DPTTGKFSCQVRGIYFFTYHILMRGGDGTSMWADL
C1ql3 DPTTGKFTCSIPGIYFFTYHVLMRGGDGTSMWADL
C1ql4 EAASGKFTCPMPGVYFFAYHVLMRGGDGTSMWADL
C1ql1 CKNGQVRASAIAQDADQNYDYASNSVILHLDAGDE
C1ql2 CKNGQVRASAIAQDADQNYDYASNSVVLHLDSGDE
C1ql3 CKNNQVRASAIAQDADQNYDYASNSVVLHLEPGDE
C1ql4 MKNGQVRASAIAQDADQNYDYASNSVILHLDVGDE
C1ql1 VFIKLDGGKAHGGNSNKYSTFSGFIIYSD
C1ql2 VYVKLDGGKAHGGNNNKYSTFSGFLLYPD
C1ql3 VYIKLDGGKAHGGNNNKYSTFSGFIIYAD
C1ql4 VFIKLDGGKVHGGNTNKYSTFSGFIIYPD
e
Comparison BAI3 vs.BAI2 NTD
BAI3 NTD
vs.
BAI2 NTD
N
N
C
C
90°
RMSD:1.1 Å
f
BAI3/C1ql3 vs. BAI2/C1ql3 interface
D190
L178
W188
C1ql3
Y244
D232
Y210
T65
C1ql3
N242
G238
BAI3 NTD
T63
D61
BAI2 NTD
Y67
K66
D63
N126
Y65
g
Evolutionary conservation of the BAI NTD
Zebraf. BAI1 SSSAFP-----SMFCTWTLQNPDPRRYTIFIKVTKPTRDCIA-RQHRTFQFDSFLETTRTF-LGMES--------------------FDEVVKLC
Bovine BAI1 AAAVFP---ANASRCSWTLRNPDPRRYTLYMKVAKAP-ACSGPARVRTYQFDSFLESTRTY-LGVES--------------------FDEVLRLC
Rat BAI1 AAAVFP---ANASRCSWTLRNPDPRRYTLYMKVAKAPAPCSGPGRVRTYQFDSFLESTRTY-LGVES--------------------FDEVLRLC
Mouse BAI1 AAAVFP---ANASRCSWTLRNPDPRRYTLYMKVAKAPAPCSGPGRVRTYQFDSFLESTRTY-LGVES--------------------FDEVLRLC
Human BAI1 AAAVFP---ANASRCSWTLRNPDPRRYTLYMKVAKAPVPCSGPGRVRTYQFDSFLESTRTY-LGVES--------------------FDEVLRLC
Zebraf. BAI2 LRDLFPSPSALASGCSWTVENPDPTKYSLYFRFNRHAGACLA-FSPLVLPLDHYLSNQTCGPPDQPSFPS------------------DPEAVELC
Bovine BAI2 LQDLFP---TIASGCSWTLENPDPTKYSLYLRFNRQEQVCTH-FAPRLLPLDHYLVNFTCLRPSPEEAEAQAGSELGRPEEEKEEE-EAAGLELC
Rat BAI2 LQDLFP---TIASGCSWTLENPDPTKYSLYLRFNRQEQVCTH-FAPRLLPLDHYLVNFTCLRPGPEEATARAESEVGRPEEEEAE-AEAAATGLELC
Mouse BAI2 LQDLFP---TIASGCSWTLENPDPTKYSLYLRFNRQEQVCTH-FAPRLLPLDHYLVNFTCLRPGPEEATARAESEVGRPEEEEEEEAAAAASGLELC
Human BAI2 LQDLFP---TIASGCSWTLENPDPTKYSLYLRFNRQEQVCAH-FAPRLLPLDHYLVNFTCLRPSPEEAVAQAESEVGRPEEEEAE---AAAGLELC
Zebraf. BAI3 MSEMFP---KNFTNCTWTLENPDPTKYSIYLKLYKREISCSE-FSLLAYQFDHFSHEKINELL---------------------------RINESIVHLC
Rat BAI3 VSEMFP---KNFTNCTWTLENPDPTKYSIYLKFSKKDLSCSN-FSLLAYQFDHFSHEKIKDLL---------------------------RKNHSIMQLC
Mouse BAI3 VSEMFP---KNFTNCTWTLENPDPTKYSIYLKFSKKDLSCSN-FSLLAYQFDHFSHEKIKDLL---------------------------RKNHSIMQLC
Human BAI3 VSEMFP---KNFTNCTWTLENPDPTKYSIYLKFSKKDLSCSN-FSLLAYQFDHFSHEKIKDLL---------------------------RKNHSIMQLC

<u>Supplementary Figure 2:</u> Electron density map for C1ql3-BAI3 complex and superposition of BAI3 NTD with CUB domains.

a. The electric potential map (grey mesh, contoured at 7.5 σ) reveals well resolved density for residue sidechain involved in C1ql3-BAI3 interaction.

b. Structure superposition of BAI3 NTD, colored in cyan, with Alpha Fold predicted BAI3 NTD structure, colored in yellow.

c. Sequence alignment of the mouse BAI1, BAI2 and BAI3 NTD domains. Conserved residues are colored blue. Disulfide bonded cysteines are identified by linked lines. The key residues that mediate C1ql3 interaction are shown by a red box.

d. Sequence alignment of C1q domains of mouse C1ql1, 2, 3 and 4. Conserved residues are colored in blue. Residues involved in BAI3 interaction are marked by asterisk.

e. Structure superposition of mouse Alpha Fold predicted BAI2 NTD (light orange) to BAI3 NTD (cyan).

f. Detailed C1ql3-BAI NTD interaction with Alpha Fold predicted BAI2 NTD (light orange) superposed to BAI3 NTD (cyan). The interacting loop residues are conserved between BAI2 and BAI3.

g. Sequence alignment of BAI NTDs from indicated species. The C1ql-interacting loops are indicated by a red box, with arrows showing mutations at positions 60 and 65. The acidic insert of BAI2 is shown by orange box. The green box indicates the helix region outside the core β sheets.

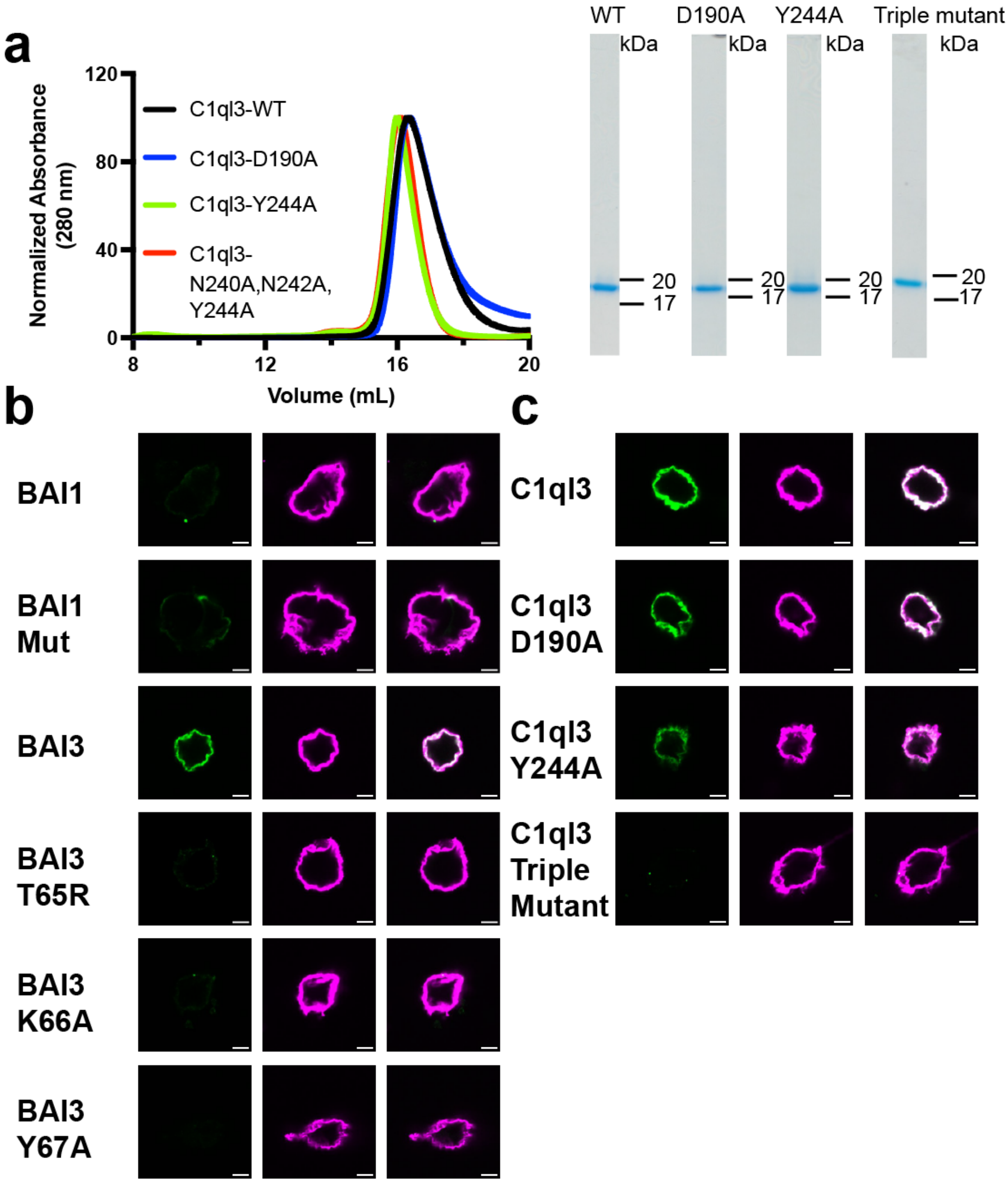

<u>Supplementary Figure 3:</u> **Interface mutations disrupt C1ql3-BAI3 interaction.**

a. HA tagged C1ql3 and C1ql3 protein showed similar size exclusion chromatography profile (left) and molecular weight by SDS-PAGE gel staining (right).

b. Cell surface labelling assay to test HA-tagged C1ql3 (green, left) binding to FLAG-tagged BAI1 and BAI3 and corresponding mutant (magenta, middle) receptors (merged, right).

c. Cell surface labelling assay to test HA tagged C1ql3 and mutant (green, left) binding to FLAG-tagged BAI3 (magenta, middle) receptors (merged, right). Scale bars (b and c) represent 5 μm.

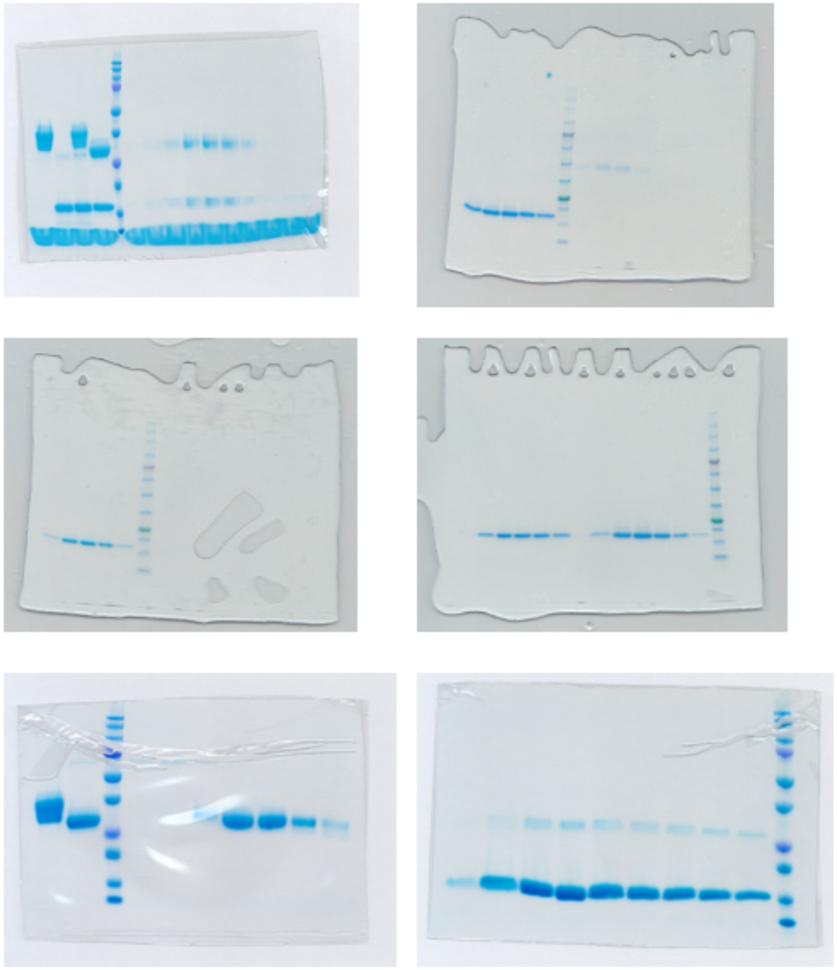

<u>**Supplementary Fig. 4:**</u> **Uncropped gels for SDS-PAGE.**

Uncropped scanned SDS-PAGE gel images.
