# Supplementary material for: Structure of the complex of C1q-like 3 protein with adhesion-GPCR BAI3
Source: Commun Biol. 2025 May 3;8:693. doi: 10.1038/s42003-025-08112-w (PMC12048575; doi:10.1038/s42003-025-08112-w)
Supplement: Supplementary file 3 — Description of Additional Supplementary Files [file 42003_2025_8112_MOESM3_ESM.pdf]

Description of Additional Supplementary Files

File name: Supplementary Data 1
Description: Raw data for size exclusion chromatography
